# 1240. Perceptions of Antimicrobial Stewardship and Antibiotic Use by Healthcare Workers in Latin America

**DOI:** 10.1093/ofid/ofad500.1080

**Published:** 2023-11-27

**Authors:** Valeria Fabre, Sara E Cosgrove, Fernanda C Lessa, Twisha S Patel, Payal K Patel, Rodolfo E Quirós

**Affiliations:** Johns Hopkins University School of Medicine, Baltimore, Maryland; Johns Hopkins School of Medicine, Baltimore, MD; CDC, Atlanta, Georgia; Chenega Enterprise Systems and Solutions (CHESS) / Centers for Disease Control and Prevention, Ann Arbor, MI; Intermountain Health, Salt Lake City, Utah; PAHO, CABA, Ciudad Autonoma de Buenos Aires, Argentina

## Abstract

**Background:**

The burden of antimicrobial resistance (AMR) in Latin America is high. Little is known about healthcare workers’ (HCWs) perceptions of antimicrobial stewardship (AS) and antibiotic use (AU) in the region.

**Methods:**

HCWs from 42 hospitals in Panama, Guatemala, Colombia, Ecuador, and Argentina were invited to participate in an electronic, voluntary, anonymous survey regarding perceptions of AS and AU from March-April 2023. All participants were asked 21 questions; prescribers were asked additional questions about antibiotic prescribing. Answers with 5-point Likert scale were categorized into two groups, strongly agree/agree and neutral/disagree/strongly disagree.

**Results:**

Of 475 HCWs that completed the survey, 52% were physicians (29% in training), 28% nurses, 13% pharmacists, 3% microbiologists, and 3% “other.” Median years of experience was 12 (interquartile range 6, 20). Although 93% indicated optimizing AU was a priority at their healthcare facility (HCF), only 69% said the importance of AS was communicated at their HCF. Nurses and those in “other” roles were more likely to report lack of familiarity with the term AS than physicians (32% and 27% respectively vs. 21%, *P*< 0.01).

Most (99%) respondents acknowledged that appropriate AU can reduce AMR and that inappropriate AU could harm patients. However, fewer thought antibiotics were overused (30%) or AMR was a problem (52%) in their HCF. Thirty-eight percent of HCWs did not have access to guidelines and 24% did not value recommendations by the AS team (**Figure 1**). Of prescribers, 99% reported to modify antibiotics based on culture results, 55% do not consult non-physician staff (e.g., pharmacists, nurses) to make antibiotic decisions, 20% do not use local guidelines or do not receive training on how to interpret culture results to make antibiotic decisions (**Figure 2**). Prescribers felt pressure from colleagues (38%) and patients or their families (63%) to make antibiotic decisions.

**Figure:**
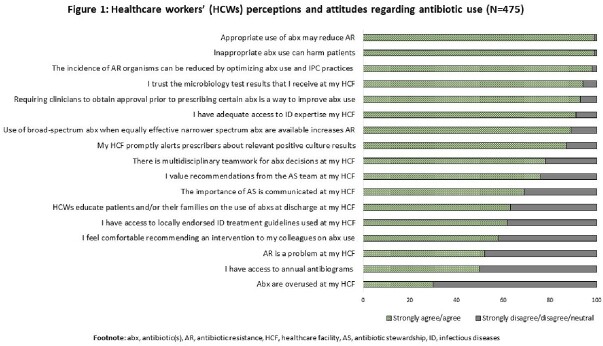

**Figure:**
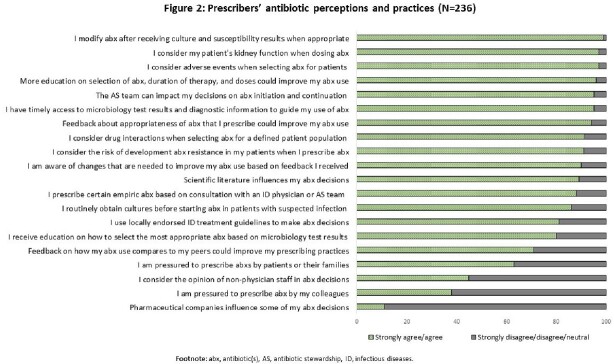

**Conclusion:**

Although most HCWs perceived improving AU as a priority, they did not perceive AU or AMR to be a problem in their HCF. Opportunities to optimize AU include improved access and adherence to guidelines, access to AMR data, teamwork, and education on AS for HCW.

**Disclosures:**

**Sara E. Cosgrove, MD, MS**, Debiopharm: Advisor/Consultant|Duke Clinical Research Institute: Advisor/Consultant **Payal K. Patel, MD MPH**, qiagen: Honoraria

